# Dose-modified lenalidomide induces sustained hematological response in patients with intermediate to high risk myelodysplasia

**DOI:** 10.4084/MJHID.2010.012

**Published:** 2010-05-31

**Authors:** Alhossain Khalafallah, Terry Brain

**Affiliations:** 1 Launceston General Hospital, Tasmania, Australia; 2 School of Human Life Sciences, University of Tasmania, Australia

**Dear Editor,**

Treating intermediate to high-risk Myelodysplastic syndrome (MDS) remains a challenge despite the availability of new agents, such as lenalidomide, azacitidine, and decitabine. Thus, immunomodulating agents, especially lenalidomide, represent an exciting and promising area of development in the treatment of MDS. Dose-escalation of lenalidomide in treating MDS and other hematologic malignancies is associated with apparent side-effects due to hemopoietic suppression. Therefore, it is worthwhile to study different dosing regimens of lenalidomide and their effect on the hematopoiesis of intermediate to high risk MDS patients.

MDS is a clonal heterogeneous hematopoietic stem cell disorder characterized by ineffective hematopoiesis.^1^ Successful treatment of MDS aims to decrease transfusion dependency, improve patient survival and quality of life, as well as decreasing the likelihood of transformation to acute myeloid leukemia (AML).^1^

Lenalidomide is an immunomodulating agent from the immunomodulatory compounds (IMiDs) class that is employed in the management of several hematological malignancies. It has been approved for the treatment of multiple myeloma and low-risk myelodysplasia (MDS), particularly in those patients with the cytogenetic abnormality; deletion of the long arm of chromosome 5 (5q-).^2^–^3^

Hematologic and cytogenetic responses achieved with lenalidomide in MDS suggest that lenalidomide controls the abnormal MDS clone in low-risk disease with a lack of data in other disease stages.^4^–^6^ The lenalidomide dose in these trials was limited to 5–10 mg per day due to drug-related myelosuppression.^7^,^8^ Moreover, low-dose lenalidomide results in a lower plasma concentration of the drug compared with higher dosing strategies.^7^–^9^ Most of these studies report that lenalidomide was administered in a dose of 5–10 mg daily for 21 days of a monthly cycle to avoid myelosuppression. There is no available data regarding dividing the monthly dose of lenalidomide (total dose: 105–210 mg) evenly into two doses of 15 mg per week (total dose: 120 mg monthly) in order to improve patient tolerance.

We describe two patients with MDS who were commenced on lenalidomide therapy between April 2009 and January 2010. Before lenalidomide treatment, both patients required regular blood transfusions every 1–4 weeks despite recombinant erythropoietin treatment. Therefore, both patients were commenced on lenalidomide therapy in addition to erythropoietin because of progressive and symptomatic anemia in conjunction with severe pancytopenia. Both patients had stable normal karyotype prior to commencement of lenalidomide therapy. They have maintained long-term transfusion independence (over 10 months in case 1 and 5 months in case 2) while receiving continuous oral lenalidomide therapy, 15 mg twice weekly. Sustained erythroid response with improvement of neutrophil and platelet counts to normal in case 2 and subnormal in case 1 with the twice weekly dose. The modified dose was commenced based on the following factors; firstly, both patients suffered from severe pancytopenia, therefore the daily dose of lenalidomide may have an undesirable effect on myelopoiesis or megakaryopoiesis. Secondly, a relatively higher dose of 15 mg twice weekly lenalidomide will theoretically achieve a higher plasma concentration of lenalidomide on the days of administration, which may have a different effect on the malignant MDS clone than does the usual daily dose of 5 mg.^7^,^9^ Lastly, the dosing regimen of lenalidomide in multiple myeloma disease incorporates a higher daily dose, between 15 mg and 25 mg, for a 21 day cycle every 28 days. Both patients consented to this treatment having refused other chemotherapeutic options. The patients were treated under the special access scheme of the Therapeutic Goods Administration, Department of Health and Aging, Australian Government.

The first patient, a 75-year-old Caucasian female was diagnosed with MDS 4 years prior to the lenalidomide therapy. The initial bone marrow biopsy showed up to 5–10 % myeloblasts expressing CD13, 33, 34 and 117 in keeping with intermediate-1 risk MDS, based on the International Prognostic Scoring System (IPSS)^10^ with no abnormal cytogenetics detected. After 2 ½ years she became transfusion-dependent at a frequency of 2–4 weeks despite erythropoietin treatment. Since commencing lenalidomide, 15 mg twice weekly, she developed a sustained erythroid response without the need for blood transfusion for 10 months thereafter.

The second patient, a 73-year-old Caucasian male with MDS was diagnosed 31 months pre-lenalidomide therapy. A bone marrow biopsy performed in March 2007 showed tri-lineage dysplasia and 5–10% myeloblasts consistent with intermediate-1 risk MDS according to IPSS. Myeloblasts were expressing CD34, 33, 117 and CD 13 in keeping with MDS. Cytogenetic analysis showed normal male karyotype. A repeat of the bone marrow biopsy 2 years later revealed an increase of the myeloblasts, up to 20%, expressing the same phenotype. At the time of commencement of lenalidomide, IPSS score was consistent with intermediate to high-risk MDS. He developed severe pancytopenia 12 months pre-lenalidomide therapy and became transfusion dependent with the requirement for blood transfusions every 1–2 weeks. Therefore, treatment with lenalidomide was initiated in August 2009. Unexpectedly, the pancytopenia recovered completely a few weeks after commencement of lenalidomide, thus eliminating his transfusion requirements thereafter with cessation of erythropietin. Although the hemoglobin (Hb) levels were maintained above 10 g/dl, the platelets and neutrophil counts dropped after 5 months of treatment but stayed above the initial levels.

There are several MDS trials employing a daily dose of lenalidomide between 5–10 mg with varying degrees of patient tolerability.^4^–^8^ However, there is no data available regarding dividing the total dose of 35 mg of lenalidomide per week into two or more doses of 15 mg weekly in order to achieve a higher concentration of lenalidomide in plasma during the days of administration and hence achieve a desirable effect on the malignant MDS clone.

Although the precise mechanism of action of lenalidomide remains unknown, it does exhibit antineoplastic and immunomodulatory properties.^2^,^3^ In patients with low-risk MDS, lenalidomide reduces the need for blood transfusions and is essential for sustained restoration of effective erythropoiesis.^6^,^7^ Lenalidomide affects the signal transduction pathway that possibly explains its selective effect on subsets of MDS and may explain the improvement of the other blood counts in the second case. While the exact molecular targets of lenalidomide remain unknown, its activity across a spectrum of neoplastic conditions highlights the possibility of multiple target sites of action.^2^,^3^

In a phase 2 trial for CLL, lenalidomide was administered at 10 mg daily with a dose-escalation of 5 mg every 28 days, however due to significant hematologic toxicity, the average delivered dose was 10 mg in this cohort of patients.^9^ This study suggested that continuous low-dose lenalidomide may be as effective as an interrupted regimen using a higher dose.^9^ However, the previous study demonstrated that the 10 mg daily dose showed significant hematologic toxicity with 30% of patients developed tumor-flare reactions that may have been attributed to the continuous daily dose of lenalidomide.

In our series, immunomodulation with lenalidomide at a dose of 15 mg twice weekly has achieved a durable hematological response in intermediate to high-risk MDS patients. Furthermore, lenalidomide has altered the disease course for these patients and has improved their quality of life without blood transfusion dependency. The drug in its modified dose has been well tolerated without side effects.

The favorable outcomes presented here show that twice weekly lenalidomide dose induced not only erythroid responses but also enhanced myelopoiesis and megakaryopoiesis as seen in case 2. However, the exact mechanism of the effect of lenalidonide in different doses on myelopoiesis and mega-karyopoiesis of MDS patients should be further studied.
